# Supplementary material for: ESKD from primary glomerulonephritis: Incidence and post KRT survival: A national cohort study in the UK
Source: PLoS One. 2026 Apr 28;21(4):e0347050. doi: 10.1371/journal.pone.0347050 (PMC13123988; doi:10.1371/journal.pone.0347050)
Supplement: S1 File — (DOCX) [file pone.0347050.s001.docx]

# **Supplementary Material**

## ERA-EDTA codes:

The following primary diagnoses / codes were included:

- IgA nephropathy – 1116, 1128, 1012
- FSGS – 1267, 1017
- Membranous nephropathy – 1185, 1014
- Mesangiocapillary glomerulonephritis – 1222, 1233, 1246, 1013, 1015
- APKD – 2718, 2725, 2739, 1041

## Supplementary Table S1: Survival cohort demographics ()

|  |  | **ADPKD** | **pGN** | **FSGS** | **IgAN** | **MN** | **MPGN** |
| --- | --- | --- | --- | --- | --- | --- | --- |
|  | N | 8302 | 10308 | 1934 | 5724 | 1636 | 1014 |
| Age First KRT | Median [IQR] | 55 (48-64) | 54 (40-67) | 55 (41-67) | 50 (37-62) | 66 (55-74) | 57 (42-69) |
|  | Range | [19-95] | [18-93] | [18-90] | [18-93] | [19-91] | [18-90] |
|  |  |  |  |  |  |  |  |
| Sex | Male | 4388 (52.9%) | 7437 (72.1%) | 1216 (62.9%) | 4410 (77%) | 1180 (72.1%) | 631 (62.2%) |
|  |  |  |  |  |  |  |  |
| Ethnicity | White | 6776 (81.6%) | 7498 (72.7%) | 1287 (66.5%) | 4247 (74.2%) | 1201 (73.4%) | 763 (75.2%) |
|  | Asian | 340 (4.1%) | 1070 (10.4%) | 216 (11.2%) | 657 (11.5%) | 127 (7.8%) | 70 (6.9%) |
|  | Black | 251 (3%) | 462 (4.5%) | 202 (10.4%) | 137 (2.4%) | 79 (4.8%) | 44 (4.3%) |
|  | Mixed | 56 (0.7%) | 92 (0.9%) | 23 (1.2%) | 54 (0.9%) | 9 (0.6%) | 6 (0.6%) |
|  | Other | 67 (0.8%) | 156 (1.5%) | 33 (1.7%) | 96 (1.7%) | 19 (1.2%) | 8 (0.8%) |
|  | Missing | {812 (10%)} | {1030 (10%)} | {173 (9%)} | {533 (9%)} | {201 (12%)} | {123 (12%)} |
| Country | England | 6621 (79.8%) | 8202 (79.6%) | 1583 (81.9%) | 4536 (79.2%) | 1256 (76.8%) | 827 (81.6%) |
|  | Scotland | 972 (11.7%) | 1199 (11.6%) | 201 (10.4%) | 683 (11.9%) | 215 (13.1%) | 100 (9.9%) |
|  | Wales | 478 (5.8%) | 681 (6.6%) | 128 (6.6%) | 365 (6.4%) | 121 (7.4%) | 67 (6.6%) |
|  | N Ireland | 231 (2.8%) | 226 (2.2%) | 22 (1.1%) | 140 (2.4%) | 44 (2.7%) | 20 (2%) |
|  |  |  |  |  |  |  |  |
| IMD Quintile | N | 6621 | 8202 | 1583 | 4536 | 1256 | 827 |
| (England Only) | Q1 | 1304 (19.7%) | 1354 (16.5%) | 243 (15.4%) | 761 (16.8%) | 204 (16.2%) | 146 (17.7%) |
|  | Q2 | 1231 (18.6%) | 1843 (22.5%) | 368 (23.2%) | 1031 (22.7%) | 253 (20.1%) | 191 (23.1%) |
|  | Q3 | 1366 (20.6%) | 1761 (21.5%) | 359 (22.7%) | 970 (21.4%) | 249 (19.8%) | 183 (22.1%) |
|  | Q4 | 1366 (20.6%) | 1713 (20.9%) | 339 (21.4%) | 916 (20.2%) | 285 (22.7%) | 173 (20.9%) |
|  | Q5 | 1354 (20.5%) | 1531 (18.7%) | 274 (17.3%) | 858 (18.9%) | 265 (21.1%) | 134 (16.2%) |
|  |  |  |  |  |  |  |  |
| Initial Treatment | Transplant | 1450 (17.5%) | 1180 (11.4%) | 199 (10.3%) | 788 (13.8%) | 90 (5.5%) | 103 (10.2%) |
|  | HD | 4376 (52.7%) | 6162 (59.8%) | 1231 (63.7%) | 3123 (54.6%) | 1161 (71%) | 647 (63.8%) |
|  | PD | 2476 (29.8%) | 2966 (28.8%) | 504 (26.1%) | 1813 (31.7%) | 385 (23.5%) | 264 (26%) |

The baseline characteristics and demographics of patients in survival cohort.

## Supplementary Table S2a: Comorbidities recorded at start of KRT – Incident Cohort

| **Comorbidity** |  | **APKD** | **pGN** | **FSGS** | **IgAN** | **MN** | **MPGN** |
| --- | --- | --- | --- | --- | --- | --- | --- |
|  | N | 3610 | 4615 | 861 | 2671 | 702 | 381 |
| **Any cardiovascular disease** | N (%) | 508 (16.7%) | 660 (16.8%) | 134 (18.3%) | 309 (13.6%) | 152 (25.1%) | 65 (19.9%) |
|  | Missing | {571 (16%)} | {679 (15%)} | {130 (15%)} | {399 (15%)} | {96 (14%)} | {54 (14%)} |
| **Chronic Obstructive Pulmonary Disease** | N (%) | 96 (2.8%) | 258 (5.9%) | 47 (5.8%) | 101 (4%) | 86 (13%) | 24 (6.6%) |
|  | Missing | {153 (4%)} | {221 (5%)} | {47 (5%)} | {118 (4%)} | {38 (5%)} | {18 (5%)} |
| **Diabetes – not causing ERF (not as Primary renal disease)** | N (%) | 208 (6.4%) | 434 (10.3%) | 96 (12.3%) | 211 (8.7%) | 93 (14.7%) | 34 (9.8%) |
|  | Missing | {338 (9%)} | {419 (9%)} | {80 (9%)} | {237 (9%)} | {69 (10%)} | {33 (9%)} |
| **Liver disease** | N (%) | 122 (3.6%) | 150 (3.4%) | 16 (2%) | 98 (3.9%) | 9 (1.4%) | 27 (7.5%) |
|  | Missing | {181 (5%)} | {249 (5%)} | {47 (5%)} | {138 (5%)} | {44 (6%)} | {20 (5%)} |
| **Malignancy** | N (%) | 197 (6.1%) | 299 (7.2%) | 56 (7.3%) | 140 (5.8%) | 78 (12.2%) | 25 (7.2%) |
|  | Missing | {386 (11%)} | {460 (10%)} | {97 (11%)} | {266 (10%)} | {61 (9%)} | {36 (9%)} |
| **Any Comorbidity** | N (%) | 942 (30.5%) | 1396 (35%) | 272 (36.7%) | 662 (29%) | 322 (51.3%) | 140 (42.3%) |
|  | Missing | {525 (15%)} | {628 (14%)} | {119 (14%)} | {385 (14%)} | {74 (11%)} | {50 (13%)} |
| **Smoking** | N (%) | 334 (11.4%) | 494 (13.2%) | 101 (14.9%) | 246 (11.4%) | 98 (16.8%) | 49 (15.3%) |
|  | Missing | {691 (19%)} | {882 (19%)} | {182 (21%)} | {521 (20%)} | {119 (17%)} | {60 (16%)} |

Baseline comorbidities recorded at the start of kidney replacement therapy (KRT) among patients in the incident cohort.

## Supplementary Table S2b: Comorbidities recorded at start of KRT – Survival Cohort

| **Comorbidity** |  | **APKD** | **pGN** | **FSGS** | **IgAN** | **MN** | **MPGN** |
| --- | --- | --- | --- | --- | --- | --- | --- |
|  | N | 5197 | 6361 | 1180 | 3596 | 981 | 604 |
| Any cardiovascular disease | N (%) | 775 (17.1%) | 950 (17%) | 190 (18.5%) | 430 (13.7%) | 224 (25.6%) | 106 (19.6%) |
|  | Missing | {672 (13%)} | {788 (12%)} | {155 (13%)} | {462 (13%)} | {107 (11%)} | {64 (11%)} |
| Chronic Obstructive Pulmonary Disease | N (%) | 144 (2.9%) | 349 (5.7%) | 76 (6.7%) | 130 (3.8%) | 104 (11.1%) | 39 (6.7%) |
|  | Missing | {182 (4%)} | {245 (4%)} | {52 (4%)} | {132 (4%)} | {41 (4%)} | {20 (3%)} |
| Diabetes – not causing ERF (not as PRD) | N (%) | 286 (6%) | 575 (9.8%) | 125 (11.6%) | 272 (8.2%) | 127 (14%) | 51 (9.1%) |
|  | Missing | {426 (8%)} | {500 (8%)} | {100 (8%)} | {282 (8%)} | {77 (8%)} | {41 (7%)} |
| Liver disease | N (%) | 150 (3%) | 188 (3.1%) | 19 (1.7%) | 115 (3.3%) | 16 (1.7%) | 38 (6.5%) |
|  | Missing | {202 (4%)} | {271 (4%)} | {51 (4%)} | {152 (4%)} | {47 (5%)} | {21 (3%)} |
| Malignancy | N (%) | 289 (6.1%) | 406 (7%) | 78 (7.3%) | 182 (5.5%) | 106 (11.6%) | 40 (7.1%) |
|  | Missing | {456 (9%)} | {533 (8%)} | {117 (10%)} | {304 (8%)} | {69 (7%)} | {43 (7%)} |
| Any Comorbidity | N (%) | 1374 (30.1%) | 1925 (34.2%) | 383 (36.9%) | 878 (27.8%) | 445 (49.6%) | 219 (40.2%) |
|  | Missing | {627 (12%)} | {729 (11%)} | {143 (12%)} | {443 (12%)} | {84 (9%)} | {59 (10%)} |
| Smoking | N (%) | 493 (11.3%) | 686 (12.9%) | 143 (14.8%) | 330 (11.1%) | 128 (15.1%) | 85 (16.3%) |
|  | Missing | {848 (16%)} | {1044 (16%)} | {213 (18%)} | {613 (17%)} | {136 (14%)} | {82 (14%)} |

Baseline comorbidities recorded at the start of kidney replacement therapy (KRT) among patients in the survival cohort.

## Supplementary Table S3: Factors associated with incidence of pGN

|  |  | **IRR (95%CI)** | |
| --- | --- | --- | --- |
|  |  | **Unadjusted** | **Adjusted** |
| Age and Sex | 18-29 M | 0.27 (0.24-0.30) | 0.27 (0.24-0.30) |
|  | 30-39 M | 0.52 (0.47-0.57) | 0.52 (0.47-0.57) |
|  | 40-49 M | 0.63 (0.58-0.69) | 0.64 (0.58-0.70) |
|  | 50-59 M | 0.84 (0.77-0.91) | 0.84 (0.77-0.91) |
|  | 60-69 M | Reference | Reference |
|  | 70-79 M | 1.21 (1.10-1.32) | 1.21 (1.10-1.32) |
|  | 80+ M | 0.77 (0.68-0.88) | 0.78 (0.68-0.89) |
|  | 18-29 F | 0.46 (0.39-0.55) | 0.46 (0.39-0.55) |
|  | 30-39 F | 0.45 (0.40-0.52) | 0.45 (0.40-0.52) |
|  | 40-49 F | 0.44 (0.39-0.49) | 0.44 (0.39-0.49) |
|  | 50-59 F | 0.35 (0.31-0.40) | 0.35 (0.31-0.40) |
|  | 60-69 F | 0.33 (0.29-0.37) | 0.33 (0.29-0.37) |
|  | 70-79 F | 0.32 (0.28-0.36) | 0.32 (0.28-0.36) |
|  | 80+ F | 0.26 (0.21-0.33) | 0.26 (0.21-0.33) |
| Country | England | Reference | Reference |
|  | N Ireland | 0.97 (0.83-1.12) | 0.98 (0.85-1.14) |
|  | Scotland | 1.26 (1.17-1.36) | 1.26 (1.16-1.36) |
|  | Wales | 1.30 (1.18-1.44) | 1.27 (1.16-1.40) |
| Deprivation  (IMD Quintile) | Q1 | Reference | Reference |
|  | Q2 | 0.96 (0.89-1.04) | 0.96 (0.89-1.04) |
|  | Q3 | 0.91 (0.85-0.99) | 0.91 (0.85-0.99) |
|  | Q4 | 0.82 (0.76-0.89) | 0.82 (0.76-0.89) |
|  | Q5 | 0.71 (0.65-0.77) | 0.71 (0.65-0.77) |

Incidence rate ratios for pGN. Unadjusted and adjusted for year, age group, sex, age by sex interaction and country. IMD is based on England-only data.

## Supplementary Table S4: Factors associated with incidence – type of pGN.

## Table S4a. IRR for each factor – IgAN

|  |  | **IRR (95%CI)** | |
| --- | --- | --- | --- |
|  |  | **Unadjusted** | **Adjusted** |
| Age and Sex | Age18-29M | 0.38 (0.33-0.43) | 0.38 (0.33-0.43) |
|  | Age30-39M | 0.72 (0.64-0.81) | 0.72 (0.64-0.81) |
|  | Age40-49M | 0.78 (0.70-0.88) | 0.79 (0.70-0.88) |
|  | Age50-59M | 0.95 (0.85-1.07) | 0.96 (0.85-1.07) |
|  | Age60-69M | Reference | Reference |
|  | Age70-79M | 1.01 (0.89-1.15) | 1.02 (0.89-1.16) |
|  | Age80+M | 0.56 (0.46-0.69) | 0.56 (0.46-0.69) |
|  | Age18-29F | 0.40 (0.32-0.49) | 0.40 (0.32-0.49) |
|  | Age30-39F | 0.37 (0.31-0.44) | 0.37 (0.31-0.44) |
|  | Age40-49F | 0.42 (0.36-0.48) | 0.42 (0.36-0.48) |
|  | Age50-59F | 0.28 (0.24-0.33) | 0.28 (0.24-0.33) |
|  | Age60-69F | 0.22 (0.18-0.27) | 0.22 (0.18-0.27) |
|  | Age70-79F | 0.17 (0.13-0.21) | 0.17 (0.13-0.21) |
|  | Age80+F | 0.16 (0.11-0.25) | 0.16 (0.11-0.24) |
| Country | England | Reference | Reference |
|  | N Ireland | 1.08 (0.90-1.29) | 1.08 (0.90-1.30) |
|  | Scotland | 1.29 (1.17-1.43) | 1.30 (1.17-1.43) |
|  | Wales | 1.20 (1.05-1.37) | 1.19 (1.04-1.36) |
| Deprivation  (IMD Quintile) | Q1 | Reference | Reference |
|  | Q2 | 0.94 (0.85-1.04) | NA (NA - NA) |
|  | Q3 | 0.87 (0.79-0.97) | NA (NA- NA) |
|  | Q4 | 0.82 (0.74-0.91) | NA (NA- NA) |
|  | Q5 | 0.71 (0.63-0.79) | NA (NA- NA) |

Incidence rate ratios for IgAN Unadjusted and adjusted for year, age group, sex, age by sex interaction and country. IMD is based on England-only data.

Table S4b. IRR for each factor – FSGS

|  |  | **IRR (95%CI)** | |
| --- | --- | --- | --- |
|  |  | **Unadjusted** | **Adjusted** |
| Age and Sex | Age18-29M | 0.16 (0.11-0.22) | 0.16 (0.12-0.22) |
|  | Age30-39M | 0.36 (0.28-0.47) | 0.37 (0.29-0.47) |
|  | Age40-49M | 0.52 (0.42-0.65) | 0.52 (0.42-0.65) |
|  | Age50-59M | 0.81 (0.66-0.99) | 0.81 (0.67-0.99) |
|  | Age60-69M | Reference | Reference |
|  | Age70-79M | 1.06 (0.86-1.32) | 1.06 (0.86-1.32) |
|  | Age80+M | 0.82 (0.61-1.10) | 0.82 (0.61-1.10) |
|  | Age18-29F | 0.89 (0.59-1.35) | 0.89 (0.59-1.35) |
|  | Age30-39F | 0.89 (0.66-1.20) | 0.89 (0.66-1.20) |
|  | Age40-49F | 0.61 (0.46-0.80) | 0.61 (0.46-0.80) |
|  | Age50-59F | 0.50 (0.39-0.63) | 0.50 (0.39-0.63) |
|  | Age60-69F | 0.54 (0.43-0.68) | 0.54 (0.43-0.68) |
|  | Age70-79F | 0.49 (0.37-0.64) | 0.49 (0.37-0.64) |
|  | Age80+F | 0.25 (0.16-0.41) | 0.25 (0.16-0.41) |
| Country | England | Reference | Reference |
|  | N ireland | 0.45 (0.28-0.72) | 0.45 (0.28-0.73) |
|  | Scotland | 1.11 (0.92-1.33) | 1.09 (0.91-1.31) |
|  | Wales | 1.38 (1.12-1.71) | 1.34 (1.08-1.66) |
| Deprivation  (IMD Quintile) | Q1 | Reference | Reference |
|  | Q2 | 0.96 (0.81-1.13) | NA (NA - NA) |
|  | Q3 | 0.89 (0.75-1.06) | NA (NA - NA) |
|  | Q4 | 0.77 (0.64-0.92) | NA (NA - NA) |
|  | Q5 | 0.67 (0.56-0.81) | NA (NA - NA) |

Incidence rate ratios for FSGS. Unadjusted and adjusted for year, age group, sex, age by sex interaction and country. IMD is based on England-only data.

Table S4c. IRR for each factor – MN

|  |  | **IRR (95%CI)** | |
| --- | --- | --- | --- |
|  |  | **Unadjusted** | **Adjusted** |
| Age and Sex | Age18-29M | 0.04 (0.02-0.07) | 0.04 (0.02-0.07) |
|  | Age30-39M | 0.12 (0.08-0.17) | 0.12 (0.08-0.17) |
|  | Age40-49M | 0.31 (0.24-0.40) | 0.31 (0.24-0.41) |
|  | Age50-59M | 0.53 (0.42-0.66) | 0.53 (0.42-0.66) |
|  | Age60-69M | Reference | Reference |
|  | Age70-79M | 1.74 (1.44-2.11) | 1.75 (1.45-2.11) |
|  | Age80+M | 1.31 (1.02-1.68) | 1.32 (1.03-1.69) |
|  | Age18-29F | 0.47 (0.18-1.24) | 0.47 (0.18-1.24) |
|  | Age30-39F | 0.31 (0.15-0.65) | 0.31 (0.15-0.65) |
|  | Age40-49F | 0.27 (0.17-0.43) | 0.27 (0.17-0.43) |
|  | Age50-59F | 0.40 (0.29-0.55) | 0.40 (0.29-0.55) |
|  | Age60-69F | 0.30 (0.23-0.40) | 0.30 (0.23-0.40) |
|  | Age70-79F | 0.40 (0.31-0.50) | 0.39 (0.31-0.50) |
|  | Age80+F | 0.29 (0.20-0.41) | 0.28 (0.20-0.41) |
| Country | England | Reference | Reference |
|  | N Ireland | 1.24 (0.88-1.73) | 1.30 (0.92-1.82) |
|  | Scotland | 1.36 (1.12-1.65) | 1.34 (1.11-1.63) |
|  | Wales | 1.41 (1.11-1.80) | 1.31 (1.03-1.68) |
| Deprivation  (IMD Quintile) | Q1 | Reference | Reference |
|  | Q2 | 0.98 (0.80-1.21) | NA (NA - NA) |
|  | Q3 | 1.10 (0.89-1.35) | NA (NA - NA) |
|  | Q4 | 1.02 (0.83-1.26) | NA (NA - NA) |
|  | Q5 | 0.78 (0.62-0.97) | NA (NA - NA) |

Incidence rate ratios for MN. Unadjusted and adjusted for year, age group, sex, age by sex interaction and country. IMD is based on England-only data.

Table S4d. IRR for each factor – MPGN

|  |  | IRR (95%CI) | |
| --- | --- | --- | --- |
|  |  | Unadjusted | Adjusted |
| Age and Sex | Age18-29M | 0.40 (0.27-0.60) | 0.40 (0.27-0.60) |
|  | Age30-39M | 0.46 (0.30-0.68) | 0.46 (0.31-0.69) |
|  | Age40-49M | 0.67 (0.47-0.95) | 0.67 (0.47-0.96) |
|  | Age50-59M | 0.85 (0.60-1.20) | 0.85 (0.60-1.20) |
|  | Age60-69M | Reference | Reference |
|  | Age70-79M | 1.70 (1.22-2.38) | 1.70 (1.22-2.38) |
|  | Age80+M | 0.86 (0.51-1.43) | 0.86 (0.51-1.43) |
|  | Age18-29F | 0.51 (0.30-0.87) | 0.51 (0.30-0.87) |
|  | Age30-39F | 0.62 (0.37-1.04) | 0.62 (0.37-1.04) |
|  | Age40-49F | 0.50 (0.32-0.78) | 0.50 (0.32-0.78) |
|  | Age50-59F | 0.58 (0.39-0.86) | 0.58 (0.39-0.86) |
|  | Age60-69F | 0.70 (0.48-1.02) | 0.70 (0.48-1.02) |
|  | Age70-79F | 0.55 (0.38-0.79) | 0.55 (0.38-0.79) |
|  | Age80+F | 0.78 (0.42-1.42) | 0.78 (0.42-1.42) |
| Country | England | Reference | Reference |
|  | N Ireland | 0.97 (0.58-1.63) | 0.99 (0.59-1.66) |
|  | Scotland | 1.22 (0.93-1.61) | 1.21 (0.92-1.59) |
|  | Wales | 1.66 (1.22-2.26) | 1.61 (1.18-2.18) |
| Deprivation  (IMD Quintile) | Q1 | Reference | Reference |
|  | Q2 | 1.06 (0.81-1.38) | NA (NA - NA) |
|  | Q3 | 0.99 (0.76-1.30) | NA (NA- NA) |
|  | Q4 | 0.66 (0.49-0.90) | NA (NA- NA) |
|  | Q5 | 0.66 (0.49-0.90) | NA (NA- NA) |

Incidence rate ratios for MPGN. Unadjusted and adjusted for year, age group, sex, age by sex interaction and country. IMD is based on England-only data.

## Supplementary Table S5 – Time to transplant

|  |  | **APKD** | **pGN** | **FSGS** | **IgAN** | **MN** | **MPGN** |
| --- | --- | --- | --- | --- | --- | --- | --- |
| Initial Tx |  | 1245 (22.8%) | 972 (13.5%) | 159 (11.4%) | 675 (16.3%) | 68 (6.3%) | 70 (11.9%) |
| Delayed Tx |  | 2414 (44.1%) | 3245 (45%) | 608 (43.7%) | 2166 (52.2%) | 278 (25.7%) | 193 (32.9%) |
| No Tx |  | 1812 (33.1%) | 2989 (41.5%) | 624 (44.9%) | 1306 (31.5%) | 736 (68%) | 323 (55.1%) |
| Time to Tx (d) | Median [IQR] | 265 (0-799) | 383 (36-888) | 555 (72-1070) | 339 (21-829) | 623 (97-1080) | 312 (0-838) |
| (for delayed) | Range | [1-4062] | [1-4740] | [2-4740] | [1-3822] | [1-3505] | [35-3000] |

Numbers with first KRT being a (pre-emptive) transplant, with a delayed transplant after other KRT and with no transplant recorded (to last follow-up) along with time from KRT to first transplant in those with a delayed transplant

*Note: 7 patients had a Tx on the first day of KRT but had PD/HD as their initial treatment – these are included as delayed transplants*

## Supplementary Table S6: Fitted survival by Age for each group

| **Group** | **KRT Age** | **1 Years** | **5 Years** | **10 Years** |
| --- | --- | --- | --- | --- |
| APKD | 18-29 | 96.7 (90.6-100.0) | 94.7 (87.6-100.0) | 92.2 (83.9-100.0) |
| APKD | 30-39 | 99.3 (98.0-100.0) | 96.8 (94.6- 99.1) | 95.3 (92.6- 98.0) |
| APKD | 40-49 | 99.7 (99.2-100.0) | 98.0 (97.2- 98.9) | 95.1 (93.8- 96.5) |
| APKD | 50-59 | 99.5 (98.9-100.0) | 96.2 (95.1- 97.3) | 87.6 (85.7- 89.5) |
| APKD | 60-69 | 98.3 (97.0- 99.6) | 88.6 (86.1- 91.1) | 74.6 (71.2- 78.1) |
| PKD | 70+ | 97.6 (94.3-100.0) | 81.4 (74.3- 89.1) | 46.4 (35.3- 61.1) |
| pGN | 18-29 | 100.0 (100.0-100.0) | 99.6 (99.2-100.0) | 98.1 (96.8-99.3) |
| pGN | 30-39 | 100.0 (100.0-100.0) | 98.7 (98.0- 99.5) | 95.3 (93.7-96.9) |
| pGN | 40-49 | 99.6 (99.0-100.0) | 97.7 (96.6- 98.7) | 93.9 (92.3-95.6) |
| pGN | 50-59 | 98.0 (96.5- 99.5) | 92.4 (90.4- 94.5) | 81.8 (78.9-84.7) |
| pGN | 60-69 | 98.9 (97.6-100.0) | 88.7 (85.9- 91.5) | 69.7 (65.8-73.9) |
| pGN | 70+ | 92.4 (86.3- 99.0) | 80.6 (73.1- 88.7) | 48.6 (38.9-60.8) |
| FSGS | 18-29 | 100.0 (100.0-100.0) | 98.6 (96.0-100.0) | 97.0 (93.0-100.0) |
| FSGS | 30-39 | 100.0 (100.0-100.0) | 98.4 (96.2-100.0) | 91.3 (85.9- 97.0) |
| FSGS | 40-49 | 98.3 (94.9-100.0) | 95.4 (91.2- 99.8) | 91.2 (85.8- 96.9) |
| FSGS | 50-59 | 98.7 (96.2-100.0) | 93.0 (88.7- 97.4) | 80.4 (73.9- 87.5) |
| FSGS | 60-69 | 97.7 (93.3-100.0) | 87.6 (80.9- 94.9) | 72.8 (64.3- 82.4) |
| FSGS | 70+ | 100.0 (100.0-100.0) | 88.5 (74.7-100.0) | 37.8 (19.0- 75.2) |
| IgAN | 18-29 | 100.0 (100.0-100.0) | 100.0 (100.0-100.0) | 98.4 (97.0-99.8) |
| IgAN | 30-39 | 100.0 (100.0-100.0) | 99.0 (98.3- 99.8) | 96.4 (94.8-98.1) |
| IgAN | 40-49 | 99.7 (99.2-100.0) | 98.5 (97.6- 99.5) | 94.5 (92.7-96.4) |
| IgAN | 50-59 | 97.7 (95.7- 99.7) | 92.6 (90.0- 95.2) | 83.7 (80.3-87.3) |
| IgAN | 60-69 | 99.5 (98.5-100.0) | 90.5 (87.3- 93.8) | 68.8 (63.5-74.6) |
| IgAN | 70+ | 89.6 (80.4- 99.8) | 76.8 (66.5- 88.7) | 49.2 (36.1-66.9) |
| MN | 18-29 | 100.0 (100.0-100.0) | 100.0 (100.0-100.0) | 100.0 (100.0-100.0) |
| MN | 30-39 | 100.0 (100.0-100.0) | 97.9 (93.9-100.0) | 95.3 (89.0-100.0) |
| MN | 40-49 | 100.0 (100.0-100.0) | 98.5 (95.6-100.0) | 97.1 (93.3-100.0) |
| MN | 50-59 | 97.1 (91.6-100.0) | 91.2 (84.1- 98.9) | 75.6 (66.5- 86.0) |
| MN | 60-69 | 97.2 (92.0-100.0) | 88.5 (81.3- 96.4) | 71.1 (62.2- 81.3) |
| MN | 70+ | 92.2 (78.8-100.0) | 78.7 (63.0- 98.2) | 51.3 (32.7- 80.3) |
| MPGN | 18-29 | 100.0 (100.0-100.0) | 98.6 (95.9-100.0) | 96.8 (92.5-100.0) |
| MPGN | 30-39 | 100.0 (100.0-100.0) | 96.9 (92.8-100.0) | 92.7 (85.9-100.0) |
| MPGN | 40-49 | 100.0 (100.0-100.0) | 92.6 (86.6- 99.1) | 89.7 (82.7- 97.2) |
| MPGN | 50-59 | 100.0 (100.0-100.0) | 92.1 (85.7- 99.1) | 79.2 (69.6- 90.2) |
| MPGN | 60-69 | 100.0 (100.0-100.0) | 77.3 (65.2- 91.6) | 62.7 (49.3- 79.6) |
| MPGN | 70+ | 100.0 (100.0-100.0) | 100.0 (100.0-100.0) | 71.2 (48.4-100.0) |

Overall survival stratified by disease group and age category at 1, 5, and 10 years of follow-up.

## Supplementary Table 7

Mean Transplant Incidence per year (2014-18)

| **Group** | **All Tx** | **First Tx-initial treatment** | **First Tx-delayed** | **Retransplant** |
| --- | --- | --- | --- | --- |
| APKD | 391.8 | 120 | 246.2 | 25.6 |
| pGN | 484.8 | 90.2 | 324.2 | 70.4 |
| IgAN | 314.4 | 62.6 | 210.2 | 41.6 |
| FSGS | 88.8 | 13.8 | 63.6 | 11.4 |
| MN | 43.4 | 6.6 | 31.4 | 5.4 |
| MPGN | 38.2 | 7.2 | 19 | 12 |

Mean annual incidence of kidney transplantation per year (2014–2018), by primary renal diagnosis and transplant category (all transplants, first transplant following initial or delayed treatment, and re-transplantation).

## Supplementary Figure 1: Unadjusted overall patient survival


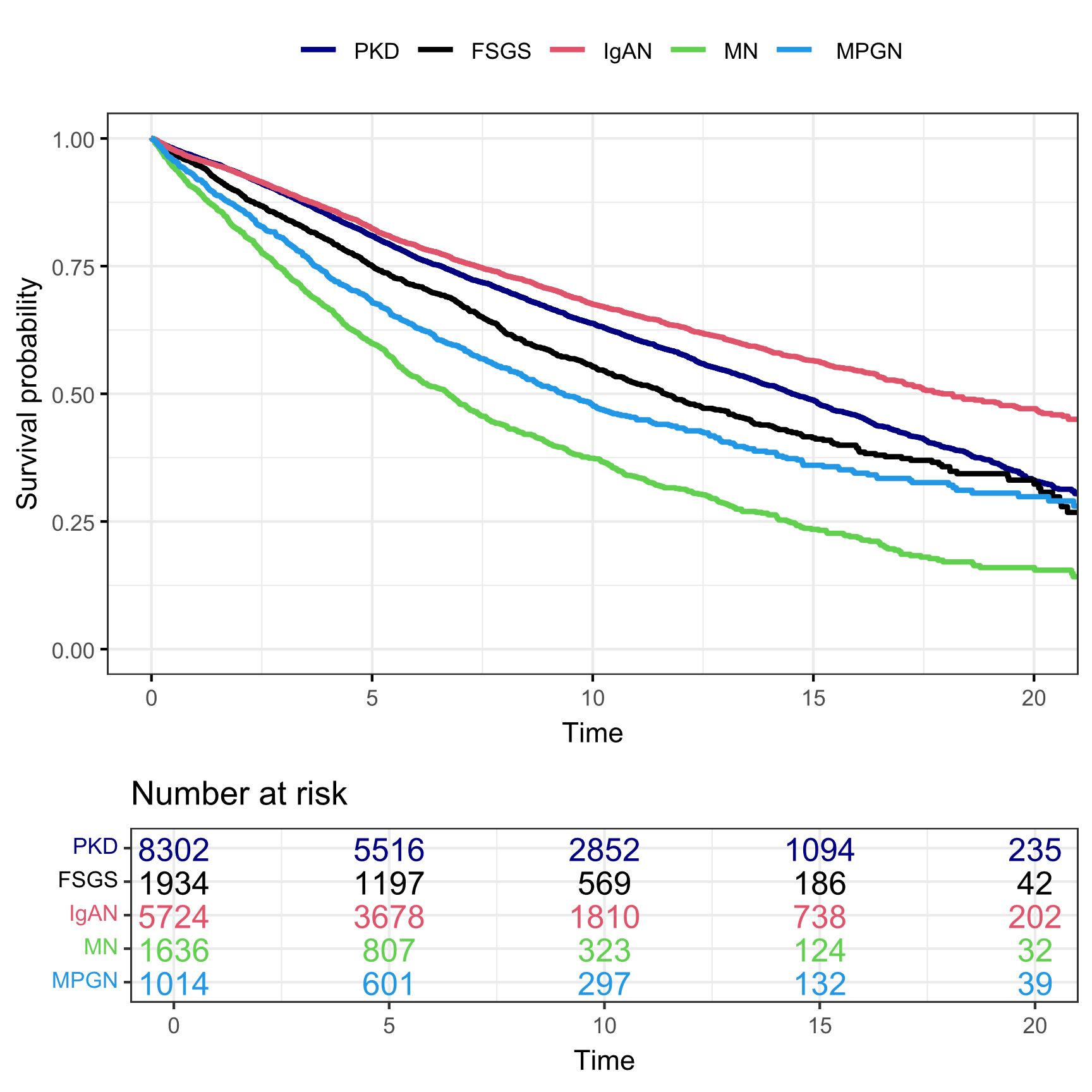


Unadjusted overall survival by disease group. Kaplan–Meier plots for four glomerulonephritis subtypes and an ADPKD comparator, with accompanying numbers-at-risk table displayed at key time intervals.

## Supplementary Figure 2: Unadjusted graft survival


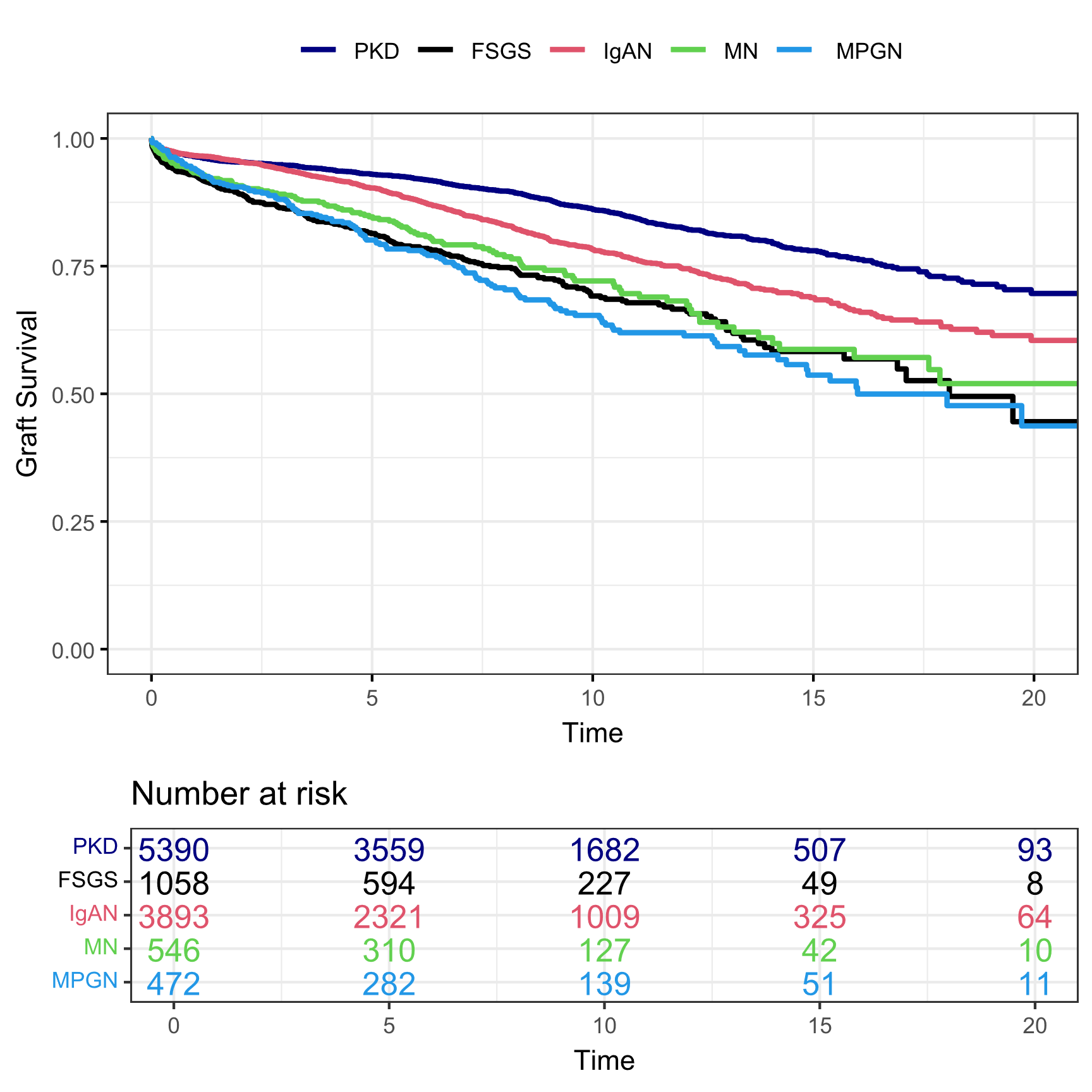


Unadjusted graft survival by disease group. Kaplan–Meier plots for four glomerulonephritis subtypes and an ADPKD comparator, with accompanying numbers-at-risk table displayed at key time intervals.

## Supplementary Figure 3: Delayed transplant

- Hazard ratio for delayed transplant 1.50 (1.28-1.76).


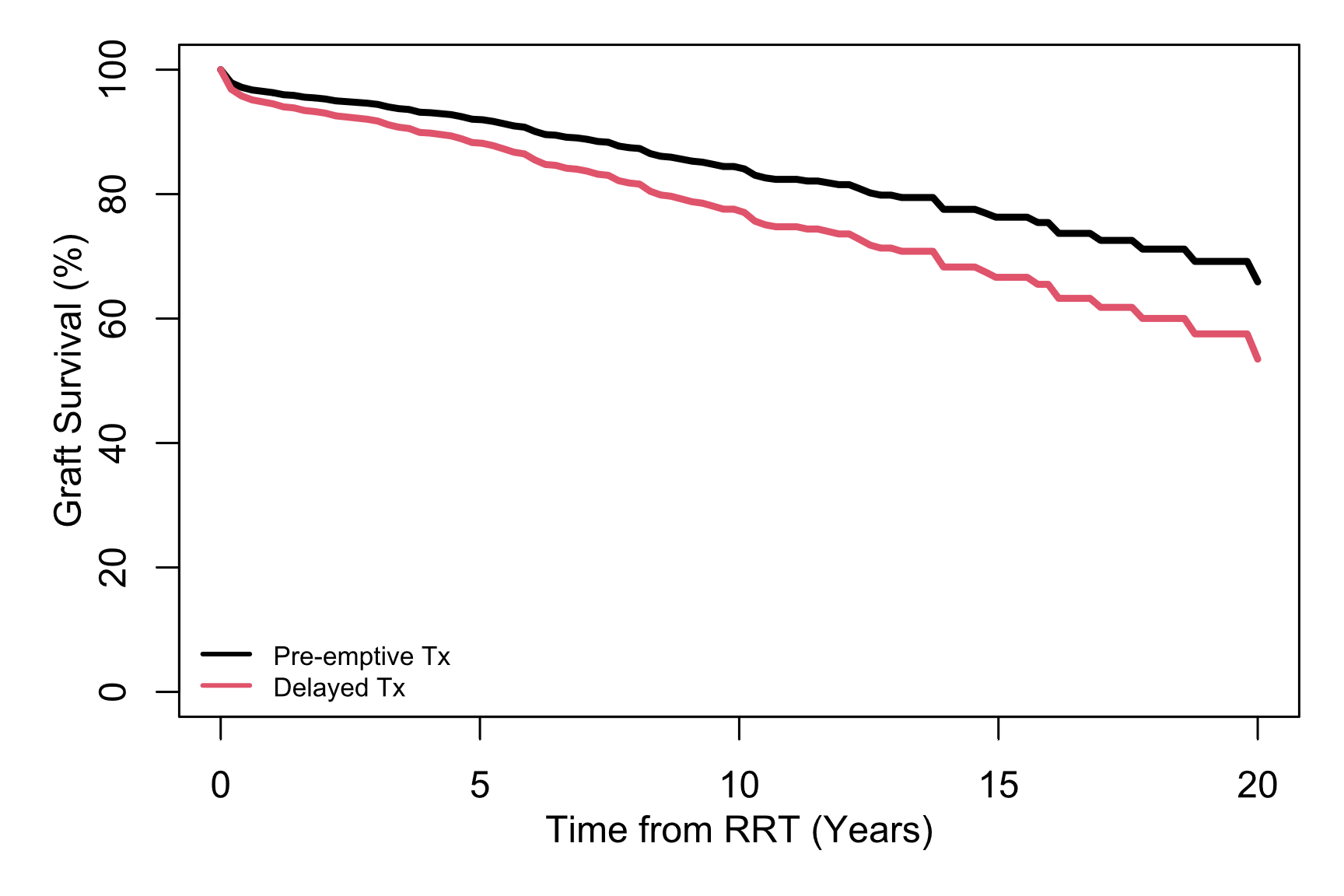


Fitted graft survival for pGN adjusting for age, sex, ethnicity as before and adding a term for whether the transplant was pre-emptive (i.e. transplant was the initial treatment recorded) or occurred after start of KRT.

## Supplementary Figure 4: Patients cohort and study timeline


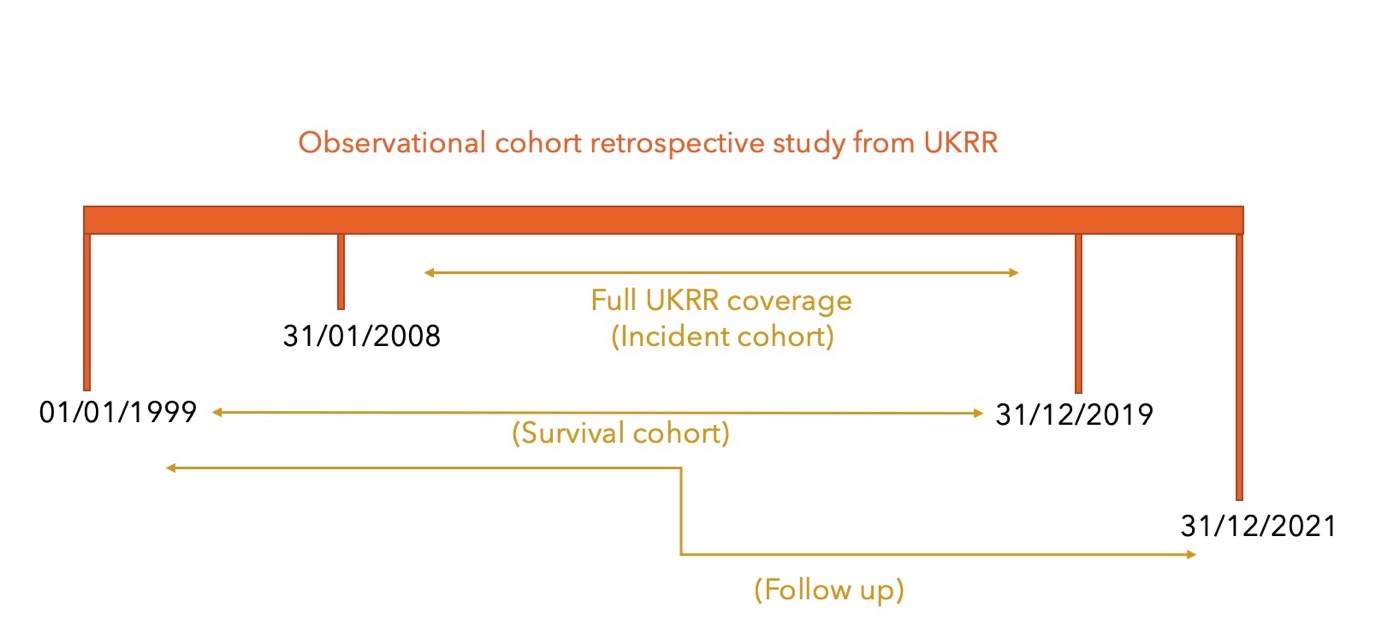


Timeline and cohort development for this observational, population-based retrospective study using UK Renal Registry (UKRR) data. Adults aged ≥18 years initiating kidney replacement therapy (KRT) in the UK were identified. The incident cohort was defined during the period of full UKRR coverage (31 January 2008 to 31 December 2019). A survival cohort was also included from an earlier timeline (01/01/1999), with follow-up extending to 31 December 2021.

## Supplementary Figure 5: Outcome definitions and censoring


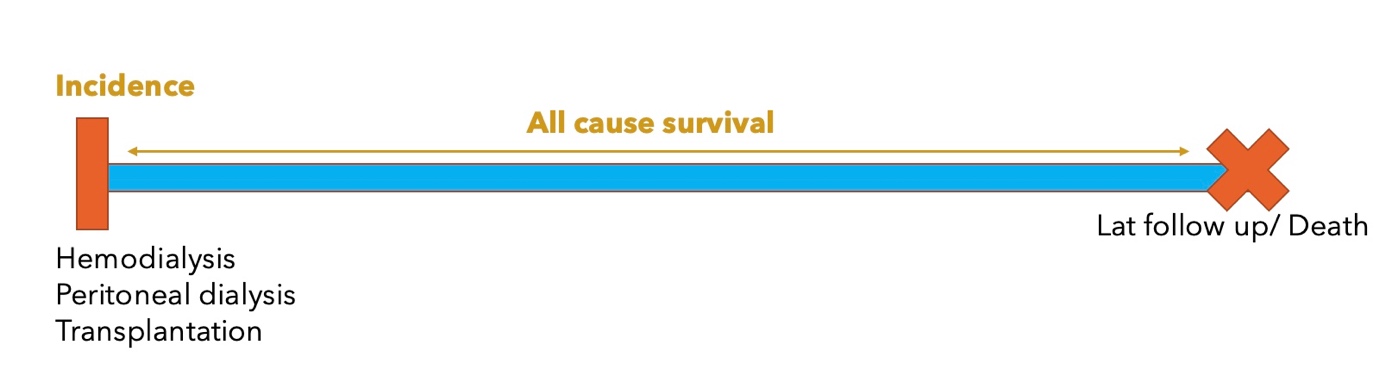


Schematic representation of outcome definitions and censoring. All-cause survival was assessed from initiation of first KRT modality (haemodialysis, peritoneal dialysis, or transplantation) until death or censoring at last follow-up.

## Supplementary Figure 6: transplant outcome definitions and censoring


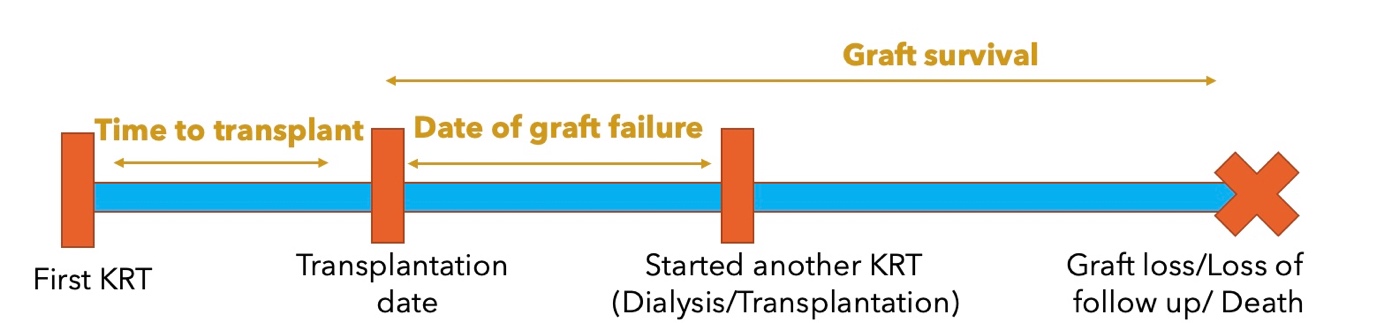


Schematic representation for transplant recipients. Graft survival was defined from transplantation date to graft failure (return to dialysis or re-transplantation), death, or loss to follow-up. Time to transplantation and subsequent KRT transitions are illustrated.

Supplementary Figure 7: Flow of patients with comorbidity and IMD data in APKD and pGN cohorts


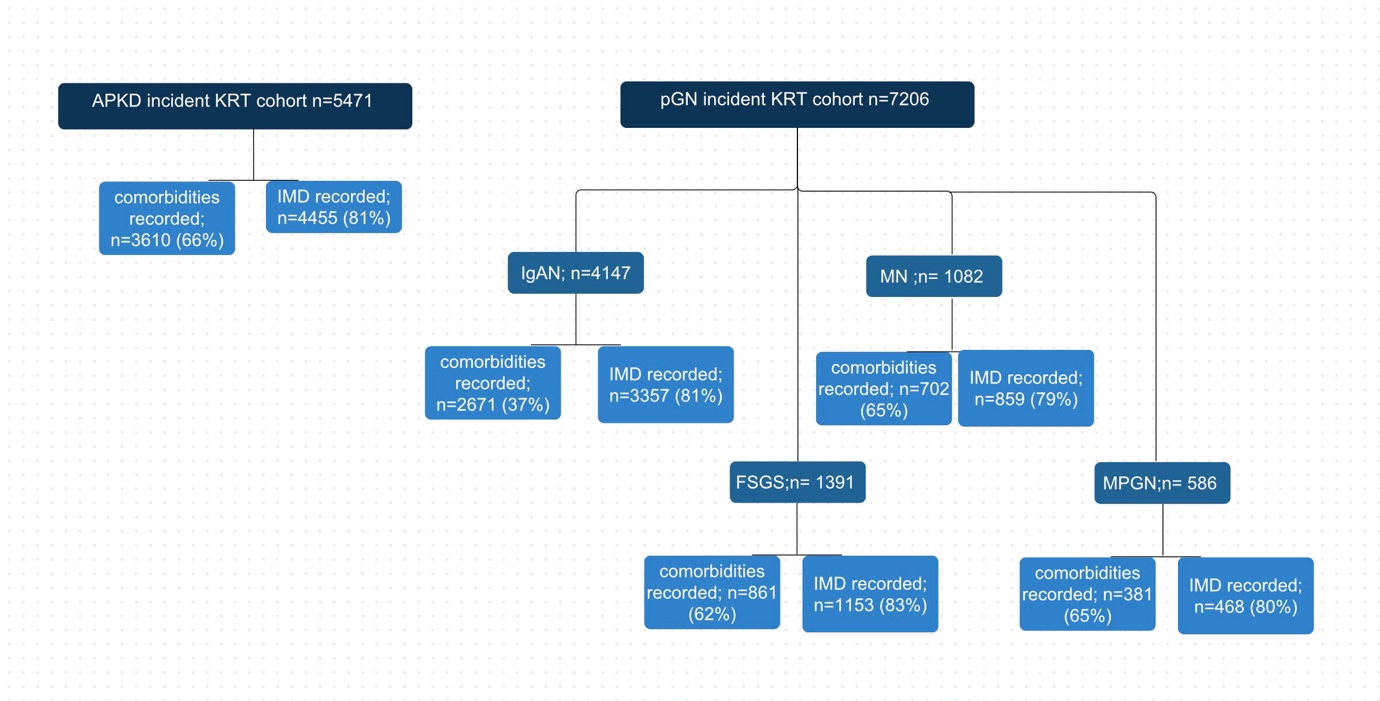


Flow diagram showing the APKD and primary glomerulonephritis (pGN) cohorts, including the number of patients with available comorbidity and IMD data for all four primary GNs.
