# Supplementary material for: ESKD from primary glomerulonephritis: Incidence and post KRT survival: A national cohort study in the UK
Source: PLoS One. 2026 Apr 28;21(4):e0347050. doi: 10.1371/journal.pone.0347050 (PMC13123988; doi:10.1371/journal.pone.0347050)
Supplement: Attachment — Submitted filename: GN_Unity_paper_Response to PLOS One .docx [file pone.0347050.s003.docx]

Incidence of ESKD and long-term outcomes in patients with primary glomerulonephritis - A UK National Cohort Study

PONE-D-25-50346

# **Reviewer's Responses to Questions**

We thank the editorial team and both reviewers for their thoughtful assessments. Where suggestions were helpful, we have incorporated changes. Where concerns were raised or based on misunderstandings, we would clarify the text and provide explanation.

**Comments to the Author**

1. Is the manuscript technically sound, and do the data support the conclusions?

The manuscript must describe a technically sound piece of scientific research with data that supports the conclusions. Experiments must have been conducted rigorously, with appropriate controls, replication, and sample sizes. The conclusions must be drawn appropriately based on the data presented.

Reviewer #1: Yes

Reviewer #2: Partly

We respectfully note that Reviewer 2 has not presented any evidence to support this assessment

2. Has the statistical analysis been performed appropriately and rigorously?

Reviewer #1: Yes

Reviewer #2: No

We strongly dispute this assessment: There are no examples presented where the analysis is questionable. Rather the reviewer seems to be looking for the results focused on P-values rather than effect sizes and their confidence intervals (this is a rather outdated presentation, as has been recommended since the seminal paper of Gardner in the BMJ in 1986 and picked up in recent years in, for example, a series of papers in Nature). The analysis and presentation follow standard practice in epidemiological studies where the aim is to describe the various populations in a comparable manner.

We also note that following the reproducible research paradigm, the statistical analysis was pre-specified in the Protocol and in a Statistical Analysis Plan which were both reviewed and agreed prior to the analysis.

3. Have the authors made all data underlying the findings in their manuscript fully available?

The [PLOS Data policy [track.editorialmanager.com]](https://urldefense.com/v3/__http:/track.editorialmanager.com/CL0/http:*2F*2Fwww.plosone.org*2Fstatic*2Fpolicies.action*23sharing/1/010f019a47e1926c-1d0d0b3e-eb0e-417c-b587-ddb5b74c122a-000000/ZAwP8M3_CEcneuBXV2OtMDGkvuJt4Z-e7FpbkH3GSFo=235__;JSUlJSU!!PDiH4ENfjr2_Jw!H8i-huJG9-fq9XKB2-6hqH5Z7VtJQPDWya9pjnR1GMC7Omk3R8tGKX3oqGdwvhIij-VmpBg202_KGDTY4s1t4INa-auft8qq-TNl5JvH$) requires authors to make all data underlying the findings described in their manuscript fully available without restriction, with rare exception (please refer to the Data Availability Statement in the manuscript PDF file). The data should be provided as part of the manuscript or its supporting information or deposited to a public repository. For example, in addition to summary statistics, the data points behind means, medians and variance measures should be available. If there are restrictions on publicly sharing data—e.g. participant privacy or use of data from a third party—those must be specified.

Reviewer #1: Yes

Reviewer #2: Yes

4. Is the manuscript presented in an intelligible fashion and written in standard English?

PLOS ONE does not copyedit accepted manuscripts, so the language in submitted articles must be clear, correct, and unambiguous. Any typographical or grammatical errors should be corrected at revision, so please note any specific errors here.

Reviewer #1: Yes

Reviewer #2: Yes

**5. Review Comments to the Author**

Please use the space provided to explain your answers to the questions above. You may also include additional comments for the author, including concerns about dual publication, research ethics, or publication ethics. (Please upload your review as an attachment if it exceeds 20,000 characters)

# **Reviewer #1**

The article is overall very informative and well written.

Some comments still are necessary to address:
1. The title does not reflect the methodology, as it does not comment on the fact that APKD was a comparator. Why was it chosen as a comparator? This is not explained in the manuscript.

We thank the reviewer for this helpful comment. We have revised the Introduction to clearly explain the rationale for including ADPKD as a comparator. ADPKD was selected because it is a common, non-immune mediated disease, well-characterised cause of ESKD with stable epidemiology, predictable natural history, and highly reliable coding within the UKRR. These features make ADPKD an ideal benchmark condition against which rarer pGNs can be meaningfully compared. In epidemiological research, crude incidence estimates alone are informative but limited, as they cannot determine whether an observed disease frequency is genuinely higher than expected. Incorporating a comparator cohort such as ADPKD allows calculation of incidence rate ratios (IRRs), strengthens causal inference by accounting for background event rates and temporal trends, and helps distinguish disease-specific risk from baseline risk in the broader ESKD population. This approach produces a more robust and clinically interpretable assessment of whether patients with pGNs experience excess risk compared with providing incidence estimates in isolation. In line 80 of the main manuscript, we cited a previous study that used the same approach to examine long-term outcomes in transplant patients, comparing those with pGNs to ADPKD.

2. It would be better to refer to APKD with the more formal name of “autosomal dominant polycystic kidney disease (ADPKD)”

Thank you for your comment. We have now adopted the full term “autosomal dominant polycystic kidney disease (ADPKD)” in the manuscript.

The language needs to be revised in several sections.

**Introduction:**
“Incidence of end stage kidney disease (ESKD) is steadily increasing worldwide, over a period of time (1, 2).” Over what period of time?
“Although considered rare in general population,” should be “in the general population”
“pGN as a group contribute” should be “contributes”

We appreciate these helpful corrections and will amend the manuscript accordingly, including:

- Clarifying the timeframe for increasing ESKD incidence. “Over the last two decades” (line 58 in main manuscript)
- “in the general population” (line 68 in main manuscript)
- “pGN as a group contributes…” (line 68 in main manuscript)

**Methods:**
The retrospective nature of the study is not pointed out in the methods.

Thank you for highlighting that. We have now clarified the retrospective nature of our study in the Methods section (line 96).

This was a retrospective cohort study using prospectively collected data from the UK Renal Registry. Patients were identified at the initiation of KRT, and outcomes were ascertained through routine registry linkage and annual reporting. We have clarified this in the Methods section.

“This was not recorded in substantial proportion and were treated as a separate category in the analyses.” Should be “in a substantial proportion” and “(this variable/ethnicity) was” “fitted survival rates and 1, 5, and 10 years” should be “fitted survival rates at 1, 5, and 10 years”

Thank you for your meticulous review and for highlighting these unintentional errors. Both have now been corrected in lines 150 and 185.

**Results:**
As the main aim is the incidence, it should be reported first.

We agree with the reviewer’s comments and have therefore added the incidence of kidney replacement therapy after the demographics section and before the patient survival outcomes.

The tables and supplemental material were not included in the provided manuscript file.

We note that the journal’s proofing system provided these as linked files. We have verified the upload and ensure all tables/supplementary files are clearly accessible in the resubmission package.
**Discussion:**
The link between migration and their impact on ethnicity in the UK therefore changing incidence of different types of pGN is not mentioned. Another factor that may have affected incidence and survival is the aging of populations.

We thank the reviewer and have added a brief comment in the Discussion (line 272-275) acknowledging demographic shifts, such as migration patterns and population ageing, as potential contributors to long-term changes in incidence and outcomes. We do, however, note that the incidence and survival models do adjust for age, sex and ethnicity.

# **Reviewer #2**

**General comments:**
1. Thanks to the authors for the work done and the interest to share with the scientific world

We thank the reviewer for their thoughtful assessments.

2. The topic biases readers to think there is discussion of primary GNs and not sure why largely authors are rather comparing to ADPKD? Was rather thinking authors can look at comparing their major causes of GNs

We thank the reviewer for raising this point. The primary purpose of the study is indeed to describe the incidence and outcomes of the four major pGN subtypes individually and collectively, the manuscript reports these extensively in tables, figures, and supplementary material.

ADPKD is included *in addition* as a stable, well-recognised reference condition to contextualise pGN estimates, not as an alternative or competing primary focus. We have clarified this rationale in the Introduction and revised the terminology to avoid misinterpretation.

We have also referenced another UK study that used a similar approach to examine long-term post-transplant outcomes in patients with primary glomerular diseases (citation 8: Pruthi R, McClure M, Casula A, Roderick PJ, Fogarty D, Harber M, et al. Long-term graft outcomes and patient survival are lower posttransplant in patients with a primary renal diagnosis of glomerulonephritis. Kidney Int. 2016;89(4):918-26.).

3. Most of the comparisons are not done analytically. Looks mainly descriptive and it might help to know of these differences are statistically significant?

We respectfully disagree. The purpose of the study is descriptive epidemiology; the central question is “What are the incidence and survival estimates?” rather than “Are the groups statistically different?”.

Accordingly, we report:

a) effect sizes (incidence rate ratios, survival estimates), and
b) 95% confidence intervals

This, following modern best practice and avoiding reliance on P-values, consistent with – Gardner & Altman’s seminal argument for effect-size reporting (BMJ 1986), and recent recommendations in Nature and leading epidemiology journals.

All regression models are fully inferential. If a reader wishes to interpret significance, they may do so using the CIs provided. We have added brief clarification in the Methods to make this philosophy explicit.

**Abstract:**
1. Authors report regression models in the methods but just reports description in the results

We agree that the incidence reporting in the abstract was brief, and we have now expanded this section. The full abstract has been revised to address the reviewer’s comments.

**Introduction:**
1. Authors report IgA nephropathy (IgAN), membranous nephropathy (MN), focal segmental glomerulosclerosis (FSGS), and membranoproliferative glomerulonephritis (MPGN) as major causes of GN in the cohort and was rather expecting analysis of these and not ADPKD?

We don’t understand this remark and presume may be related to the reviewer not being able to view the tables. The results for the 4 conditions (and GN as a group) are extensively reported in the tables provided.

2. Might help not to start sentences with Arabic numerals.

We have revised all sentences beginning with Arabic numerals throughout the manuscript to conform with standard editorial style.

**Methods:**
1. Authors should report with some short notes on the UK renal registry for the benefit of the readership

We have added a dedicated paragraph in the Methods describing the UK Renal Registry’s prospective data collection, national population coverage, diagnostic coding framework, and data quality assurance processes ( from line 117-127).

2. Line 138 - Incidence rates are normalised to the relevant age, sex and country” – not sure if authors meant standardized?

This is now line 163. We have revised the wording to “standardised” to avoid ambiguity.

3. Line 159 - “Results are presented as fitted survival rates and 1, 5, and 10 years with 95% CI” – did not appreciate a lot of the 95% confidence interval the results.

The 95% confidence interval (CI) is the standard way to present the precision of estimates. We kindly request the reviewer to re-examine Tables 3–5 and Supplementary Tables 3, 4, and 6, where all results are reported with 95% CIs.

**Results:**
1. Line 169-170 “Most patients were white (89.1% in APKD and 78.7% in pGN). In the pGN group, a significant male predominance was observed at 71.6% compared to 53.1% in APKD” – was this proven to be statistically significant?

This is a comment on the demographic table 1 and describes the sample, statistical testing of differences is not appropriate here. If the reviewer is interested a trivial chi-squared test would be highly significant given the large numbers in the cohort. In the main analyses we do show the differences in incidence between the sexes and even comment that this is significant.

2. Authors should hope to report the significant of all the analysis statistically

We chose to use a standard epidemiological presentation which focuses on effect sizes and CI. This was for 2 reasons (a) to follow standard epidemiological practice and (b) to follow the movement across medical statistics and biosciences away from a P-value centric approach with the arbitrary P<0.05 threshold to a more nuanced effect-size approach. The lead statistician has hardly published a P-value in the last 10 years across a wide range of biomedical journals.

**Discussion:**
1. Might be helpful to have looked at the GNs as per topic and not sure why authors and would have loved to see how the various GNs progress and their transplant outcomes.

We thank the reviewer. Within the constraints of available UKRR data, we have fully reported transplant outcomes for all four pGN subtypes (Figures 4, 5, Tables 3, 5, supplementary figure 2). Natural-history analyses prior to ESKD (i.e., GN progression) are unfortunately not possible using this registry data, which is derived from ESKD registry, but we note this explicitly and mention ongoing follow-up for future analyses.

We believe all the GNs are fully reported in the tables, although some of the details of the 4 conditions are relegated to supplementary tables, given the extent of the data available and prioritised the more relevant ones in the main manuscript. Follow-up of this cohort is ongoing, and we would hope as the data matures to be able to add more on the disease progress, especially the transplant related outcomes.
