# Supplementary material for: ESKD from primary glomerulonephritis: Incidence and post KRT survival: A national cohort study in the UK
Source: PLoS One. 2026 Apr 28;21(4):e0347050. doi: 10.1371/journal.pone.0347050 (PMC13123988; doi:10.1371/journal.pone.0347050)
Supplement: Attachment — Submitted filename: Letter to_Editor-Responses to Reviewers-v3-OR_DK_260214.docx [file pone.0347050.s004.docx]

Response Letter to the Editor

Dear Professor Alwahaibi,

We thank you for the opportunity to revise and resubmit our manuscript entitled “Incidence of ESKD and long-term outcomes in primary glomerulonephritis: a UK national cohort study.” We are grateful to the editorial team and the reviewers for their careful evaluation and constructive feedback, which has helped us to improve the clarity, transparency, and presentation of the manuscript.

In the revised version, we have:

a) Explicitly clarified that the study is a descriptive, population-based epidemiological analysis using national registry data.
b) Reframed autosomal dominant polycystic kidney disease (ADPKD) as a reference disease rather than a causal comparator.
c) Added a cohort flow/timeline diagram to enhance transparency and STROBE compliance.
d) Strengthened discussion of selection bias, residual confounding, and limitations related to registry-based data.
e) Harmonised incidence figures, refined language to avoid causal inference, and addressed all stylistic and formatting issues raised.

We have provided a detailed, point-by-point response to all reviewers’ and editorial comments in the accompanying document. We believe that these revisions address the concerns raised and further strengthen the manuscript, while preserving the scientific integrity of the work.

We hope that the revised manuscript is now suitable for publication in PLOS ONE and would be pleased to respond to any further queries.

Yours sincerely,

Drs Omar Ragy, Stephen Roberts and Durga AK Kanigicherla
On behalf of the authors (GN-UNITY Study Group)

# Summary of revisions

The following changes have been implemented or are to be actioned in the revised manuscript:

## 1. Abstract – KRT-incidence

a) We now used “incidence of KRT initiation due to primary glomerulonephritis rather than overall ESKD incidence.”

## 2. Introduction – descriptive intent

Added this sentence in Introduction: “This is a descriptive, population-based epidemiological study using national registry data, primarily designed to estimate the incidence of ESKD due to primary glomerulonephritis and associated long-term KRT outcomes, including modality use and patient and graft survival, rather than to test causal hypotheses.”

## 3. Methods – ADPKD as a reference disease (not a comparator)

a) Replaced ADPKD as the comparator with this wording: “ADPKD was included as a reference disease to provide contextual background for incidence and outcome estimates for a common, non–immune-mediated cause of ESKD with stable epidemiology; it was not intended as a matched control or exposure comparator, given its distinct pathogenesis, disease course, and transplantation pathways compared with primary glomerulonephritis.”
b) Added at the end of the Statistical Methods section: “Consistent with contemporary epidemiological reporting standards, analyses was focussed on estimation of effect sizes with corresponding confidence intervals rather than hypothesis testing based on P-values. Comparisons are descriptive and hypothesis-generating, and no formal adjustment for multiple comparisons was undertaken.”

## 4. Methods - missingness

a) Clarified: “Missingness was not strongly associated with calendar year or centre, although differential missingness by disease severity or socioeconomic factors cannot be excluded. Its potential impact, including for ethnicity, should be considered when interpreting subgroup estimates.”

## 5. Results / Abstract – incidence numbers

a) Added a sentence in Results to clarify: “The overall population incidence of KRT due to pGN was 11.8 per million population.”

## 6. Results – inferential language

a) Replaced “a significant male predominance was observed” with “A male predominance was observed.”

## 7. Discussion – PD and pre-emptive transplant conclusions

a) Replaced previous version with: “Peritoneal dialysis was associated with survival outcomes comparable to transplantation in selected subgroups, particularly IgAN, although these findings are observational and likely influenced by treatment selection and residual confounding. They should therefore be considered hypothesis-generating and require confirmation in prospective studies.”

## 8. Discussion – selection bias & confounding (in Limitations)

a) Expanded the limitations paragraph in Discussion and included the context: Several limitations inherent to registry-based observational studies should be considered when interpreting these findings. First, although the UK Renal Registry collects data prospectively, the present analyses were conducted retrospectively. Second, comorbidity data were available for approximately two-thirds of the cohort, and disease severity measures were not captured, limiting the ability to fully adjust for clinical risk. Third, primary renal diagnoses were based on registry coding (ERA–EDTA classifications) rather than central pathology review, and reasons for graft failure were unavailable. In addition, the MPGN category did not distinguish between primary complement-mediated disease and secondary causes such as infection or monoclonal gammopathy, which may introduce heterogeneity within this subgroup.

No formal adjustment for multiple comparisons was applied, as the analyses were descriptive and hypothesis-generating in nature. Residual confounding remains possible, including confounding by indication in dialysis modality selection and timing of transplantation. Although sensitivity analyses incorporating comorbidity data demonstrated consistent direction and magnitude of associations, incomplete covariate data and absence of detailed disease severity metrics limit causal interpretation. Accordingly, observed differences in survival should be interpreted as associations rather than evidence of treatment effect.

Furthermore, the registry captures only patients initiating kidney replacement therapy; individuals with pGN managed conservatively or with delayed progression are not included. Therefore, the reported incidence reflects KRT-incidence due to pGN rather than overall population incidence of disease. Despite these limitations, strengths of the study include nationwide coverage, high data completeness, minimal loss to follow-up, and the inclusion of ADPKD as a reference disease to contextualise incidence and outcome patterns.

## 9. Cohort flow / timeline diagram (Supplementary Figures)

a) Added cohort flow / timeline diagram in supplementary material (Supplementary Fig 4-7).

# Editorial comments

## Editorial comment 1

Please ensure that your manuscript meets PLOS ONE's style requirements, including those for file naming.

### Authors’ response 1:

We confirm that the manuscript has been carefully reviewed and revised to comply fully with PLOS ONE’s style and formatting requirements.

## Editorial comment 2

This study was supported through a research grant from CSL Vifor.

At this time, please address the following queries:

a) Please clarify the sources of funding (financial or material support) for your study. List the grants or organizations that supported your study, including funding received from your institution.

### Authors’ response 2a:

Thank you for your comment. In Line 303 we stated the following under the funding section: “This study was supported through a research grant from CSL Vifor. The company had no role in the design of the study, analysis of the data, interpretation of the results, or in the preparation of the manuscript. The views expressed are solely those of the authors.”

b) State what role the funders took in the study. If the funders had no role in your study, please state: “The funders had no role in study design, data collection and analysis, decision to publish, or preparation of the manuscript.”

### Authors’ response 2b:

Thank you for your comment. We clarified with the statement in line 302 under the funding section.

c) If any authors received a salary from any of your funders, please state which authors and which funders.

### Authors’ response 2c:

Thank you for your comment. None of the authors received a salary from the funder.

d) If you did not receive any funding for this study, please state: “The authors received no specific funding for this work.”

### Authors’ response 2d:

Thank you for requesting clarification on the study funding. We have added a sentence in line 305.

Please include your amended statements within your cover letter; we will change the online submission form on your behalf.

## Editorial comment 3

3. For studies involving third-party data, we encourage authors to share any data specific to their analyses that they can legally distribute.

PLOS recognizes, however, that authors may be using third-party data they do not have the rights to share. When third-party data cannot be publicly shared, authors must provide all information necessary for interested researchers to apply to gain access to the data. (<https://journals.plos.org/plosone/s/data-availability#loc-acceptable-data-access-restrictions>)

For any third-party data that the authors cannot legally distribute, they should include the following information in their Data Availability Statement upon submission:

1) A description of the data set and the third-party source.

2) If applicable, verification of permission to use the data set.

3) Confirmation of whether the authors received any special privileges in accessing the data that other researchers would not have.

4) All necessary contact information others would need to apply to gain access to the data.

### Authors’ response 3:

We thank the Editorial Board for the opportunity to clarify the data access arrangements.

- The data used in this study were obtained from the UK Renal Registry (UKRR) under a standard data access request, in accordance with UKRR governance procedures. The authors do not have legal authority to publicly distribute the individual-level registry data due to patient confidentiality and data protection regulations.
- The data underlying this study were obtained from the UK Renal Registry (UKRR), a national registry of patients receiving kidney replacement therapy in the United Kingdom. The dataset comprises de-identified individual-level data, including demographic characteristics, primary renal diagnosis (coded using ERA–EDTA classifications), treatment modality, transplantation events, and outcome data (including death and graft failure).
- We, as authors, accessed these data following approval of a standard data access application through the UK Renal Registry governance framework. Permission was granted for use of the dataset for the purposes of this specific research study.
- Due to legal, ethical, and data protection restrictions related to the UKRR policy, we are not permitted to publicly share the underlying individual-level data.
- We, as authors, confirm that we did not receive any special privileges in accessing the data that other qualified researchers would not have. Access is available to bona fide researchers through the UK Renal Registry’s formal data request process.
- Details regarding application procedures, governance requirements, and data access policies are available on the UKRR website. Researchers wishing to access the data may apply directly to the UK Renal Registry via: UK Renal Registry, UK Kidney Association

## Editorial comment 4

4. Please include a separate caption for each figure in your manuscript.

### Authors’ response 4:

Thank you for your comment. We have uploaded the figures again with a separate caption for each.

## Editorial comment 5

5. Please include a copy of Table 1-5 which you refer to in your text on page 6-7.

### Authors’ response 5:

Thank you for your comment. We have now included all five tables in the main manuscript as requested.

## Editorial comment 6

6. Please include captions for your Supporting Information files at the end of your manuscript, and update any in-text citations to match accordingly. Please see our Supporting Information guidelines for more information: <http://journals.plos.org/plosone/s/supporting-information>.

### Authors’ response 6:

Thank you for highlighting this point. We have now added captions for all supplementary tables/figures and uploaded as a separate file.

## Editorial comment 7

7. Please include your tables as part of your main manuscript and remove the individual files. Please note that supplementary tables (should remain/ be uploaded) as separate "Supporting Information" files.

### Authors’ response 7:

Thank you for your comment. We have now included all five tables in the main manuscript as requested and removed the individual files.

# Responses to Reviewers’ Comments

## Reviewer #3:

The authors provide descriptive findings of a study using UK registry data to evaluate incidence rates of KRT and survival following KRT initiation by the different forms of primary glomerulonephritis and/or various demographic subgroups. I believe the authors have adequately addressed the comments of the reviewers from the first review, though I have a few additional comments.

**Authors’ response:** We thank the reviewer for their careful re-evaluation of the manuscript and for recognising the revisions made in response to the initial review. We appreciate their positive assessment and are pleased that the clarifications and additional revisions have addressed the previous concerns.

1. Although PLOS One publishes epidemiologic studies of both a descriptive and etiologic nature, from research groups that publish one type of study more often than another (e.g. academic group, health agency), the wording in the early parts of the manuscript can explicitly state that it aims to describe incidence rates as a descriptive or surveillance study using national registry data, to make it more clear for readers who may be more used to etiologic studies with hypothesis testing.

**Authors’ response:** We thank the reviewer for this constructive suggestion. We agree that clearer framing will benefit readers who may be more familiar with etiologic or hypothesis-driven studies. We have revised the Introduction to explicitly state (lines 79-82) “This is a descriptive, population-based epidemiological study using national registry data, primarily designed to estimate the incidence of KRT due to primary glomerulonephritis and associated long-term KRT outcomes, including modality use and patient and graft survival, rather than to test causal hypotheses.”

2. The development of the two cohorts can also be made clearer, perhaps with a diagram akin to a STROBE diagram, but showing the timeline of the cohorts on an axis, with key points in time indicated such as the start and end of observation periods to ascertain initiation of KRT, mortality, left/right censoring, etc. I am not able to view supplementary material to see if this already exists, but I don't see a reference to a similar diagram in the text.

**Authors’ response:** We thank the reviewer for this helpful suggestion. To improve clarity and transparency, we have added a schematic diagram illustrating the construction of the incidence and survival cohorts, including the observation periods, censoring, and key time points for KRT initiation, mortality, and follow-up (Supplementary Figure 4, 5 and 6). These diagrams are provided as supplementary figures in supplementary material and referenced in the patient’s cohort and definition sections.

## Reviewer #4:

This study was based on national data from the UK Kidney Registry to analyze the epidemiological characteristics, incidence and survival outcomes of primary glomerulonephritis patients initiating renal replacement therapy from 1999 to 2019, with ADPKD as the control. The study design was reasonable, the sample size was large, the follow-up time was long, and the statistical methods were appropriate, which met the STROBE standards. The revised manuscript has complete structure, clear presentation, reliable conclusions, and clear clinical and public health value.

**Authors’ response:** We sincerely thank the reviewer for their thoughtful evaluation of our work and for recognising the strengths of the study design, dataset, and analytical approach. We appreciate their positive assessment of the manuscript’s clarity, methodological rigor, and clinical and public health relevance. We are grateful for the time and expertise invested in reviewing our work.

1. The methodological orientation of the control group was not clear ADPKD is referred to as comparator in many cases, but it is not a matched control, nor is it an expose-non-exposure design. Its pathogenesis, disease progression, and transplantation strategy are significantly different from pGN. It is recommended that ADPKD be clearly defined as a reference disease in Methods, which is used to provide background incidence and outcome reference, rather than a causal comparison object. It is clearly pointed out in Discussion and Conclusions that the difference between pGN and ADPKD cannot be explained by the disease itself.

**Authors’ response:** We thank the reviewer for this important clarification. We agree and have revised the manuscript methodology section (lines 93-97) to consistently define ADPKD as a reference disease, rather than a matched control or exposure group. Its role is to provide background incidence and outcome context for a common, non-immune mediated cause of ESKD with stable epidemiology, rather than to imply causal comparability with pGN.

2. There was limited control for potential confounders in the main analysis. Comorbidities were used only in the sub analysis, ethnicity was severely missing and was not included in the analysis, and variables related to disease severity could not be adjusted, but the main outcome still presented a deterministic survival comparison. It is recommended that these limitations be acknowledged in the Discussion system, that differences in survival may be affected by selection by indication, and that the direction of main effect consistency may be reported in subsamples with complete comorbidity data to support the robustness of the results.

**Authors’ response:** We thank the reviewer for highlighting this important limitation. We have expanded the Discussion to more clearly acknowledge residual confounding, including incomplete comorbidity data, missing ethnicity, and the absence of disease-severity measures. We now explicitly discuss selection by indication as a potential influence on survival comparisons. To support robustness, we clarify that the direction and magnitude of key associations were consistent in sensitivity analyses restricted to subsets with complete comorbidity data, although these analyses were not powered for formal subgroup inference (lines 100-102 and 283-285).

3. Follow-up and selection bias were not adequately emphasized. Only patients enrolled in KRT were included, which means that patients with mild or delayed pGN were missing, and the actual incidence was calculated as KRT-incidence rather than the overall population pGN incidence. It is recommended that this be clearly stated in the Abstract and Discussion to comply with STROBE requirements regarding study subject selection and interpretation of results.

**Authors’ response:** We agree and thank the reviewer. We have clarified throughout the Abstract (lines 25-27), Methods (lines 123-124), and that the incidence estimates represent incidence of KRT initiation due to pGN, rather than overall population incidence of ESKD-pGN. We now explicitly state that patients managed conservatively are not captured in the UKRR (Methods lines 113-115, and Discussion lines 226-227).

4. PD and pre-emptive transplant conclusions need to be lowered. This article mentioned that PD has a similar performance to transplantation in IgAN, and pre-emptive transplantation has a better outcome, but the treatment selection bias is not fully controlled, and there is a risk of causal extrapolation. It is recommended to use conditional and exploratory language and to make it clear that the findings cannot be taken as evidence to guide treatment choices and need to be validated by prospective studies.

**Authors’ response:** We thank the reviewer and agree. We have revised the Discussion to adopt more conditional and exploratory language when describing outcomes associated with peritoneal dialysis and pre-emptive transplantation. We now explicitly state that these findings are observational, subject to treatment selection bias, and should not be interpreted as evidence to guide treatment choice, but rather as hypotheses that require confirmation in prospective studies (lines 272-276).

5. STROBE report completeness The manuscript can further improve the STROBE report, and it is suggested to provide cohort flow diagram in supplementary materials and clarify the actual number of patients included in each analysis, especially in IMD and comorbidity subanalyses, to increase the transparency and reproducibility.

**Authors’ response:** We thank the reviewer. We have created a flow diagram showing the APKD and primary glomerulonephritis (pGN) cohorts, including the number of patients with available comorbidity and IMD data for all four primary GNs, and have included this as Supplementary Figure 7.

6. Handling of missing data There are many missing key variables such as race. Ethnicity can be used as the missing category, but the direction of potential bias should be explained, and a brief description of whether the correlation between the missing pattern and the outcome and its possible influence should be evaluated.

**Authors’ response:** We thank the reviewer for this important point. Ethnicity data were incomplete for a substantial proportion of patients. Ethnicity was not included in the primary incidence models due to the high proportion of missing data and limitations in available denominator population data. We have now clarified this in the Methods (lines 158-159) and the Limitations sections (line 309) to address the implications of missingness. We examined whether missing ethnicity was strongly associated with calendar year or reporting centre and did not observe marked systematic patterns. Nevertheless, differential missingness related to disease severity, socioeconomic status, or other unmeasured factors cannot be excluded. Such bias would most likely attenuate subgroup differences rather than generate spurious associations.

7. Statistical presentation Some expressions can still be misleading; for example, "significant male predominance" is easily confused with statistical significance. In addition, for questions of multiple comparisons, such as incidence or survival between different pGN subtypes and ADPKD, adjustment should be stated in the methods or limitations, and if not, the justification should be explained.

**Authors’ response:** We thank the reviewer for this helpful suggestion. We have revised terminology such as “significant” to non-inferential language “preponderance” or ‘substantial” (lines 187, 204, and 208) to avoid confusion with statistical significance. We also clarified in the Limitations that no formal adjustment for multiple comparisons was applied, as the analyses were descriptive and hypothesis-generating (lines 316-317).

8. Text and formatting problems: Some sentences in the manuscript still have redundancy, such as the first half of Introduction can be compressed; The values in the table are generally consistent with the text. Clear legends provided that all figures are correctly embedded with clear titles and notes; The reference format should be unified to ensure that the citations in the text are consistent with the references.

**Authors’ response:** We have carefully edited the manuscript to reduce redundancy in the Introduction, corrected minor formatting issues, ensured all figures are properly embedded with clear legends, and harmonised reference formatting to ensure consistency between in-text citations and the reference list.

9. Consistency of key numbers The total incidence of pGN in the abstract was "17.4 pmp", while the result of the text was "11.9 pmp" (line 247 of the text), which was inconsistent. The authors were asked to check the data and harmonize the figures to avoid confusing the readers.

**Authors’ response:** We thank the reviewer for identifying this inconsistency. We have reviewed the calculations and clarified that 17.4 pmp refers to sex-specific incidence, whereas 11.8 pmp refers to overall population incidence. The text abstract and discussion is now harmonised to clearly distinguish these estimates and avoid confusion (lines 36, 201 and 245).

10. Key findings and interpretation of clinical significance: Some of the results, such as the similar 5-year survival rate after PD and transplantation, or some differences in morbidity and survival, need to be interpreted with caution in the Discussion to avoid excessive inferences. Further exploration of potential biologic or clinical mechanisms for these unusual findings is warranted, and selection bias or unmeasured confounding may have influenced the results.

**Authors’ response:** We agree and have revised the Discussion to interpret these findings more cautiously, emphasising the potential influence of selection bias, unmeasured confounding, and registry-based limitations. We have reframed these observations as hypotheses for further investigation rather than definitive clinical conclusions (lines 271-275).
